# The value of 3D visualization operative planning system in ultrasound-guided percutaneous microwave ablation for large hepatic hemangiomas: a clinical comparative study

**DOI:** 10.1186/s12885-019-5682-5

**Published:** 2019-06-07

**Authors:** Xin Li, Chao An, Fangyi Liu, Zhigang Cheng, Zhiyu Han, Xiaoling Yu, Linan Dong, Jie Yu, Ping Liang

**Affiliations:** Department of Interventional Ultrasound, Chinese PLA General Hospital, 28 Fuxing Road, Beijing, 100853 China

**Keywords:** 3D visualization operative planning system, Microwave ablation, Large hepatic hemangiomas

## Abstract

**Background:**

To evaluate the value of a three dimension (3D)visualization operative planning system in ultrasound-guided percutaneous microwave ablation (US-PMWA) for large hepatic hemangiomas (LHHs).

**Methods:**

Fifty-eight patients with LHHs were divided into 3D and 2D groups. The therapeutic efficacy was assessed by contrast-enhanced imaging during follow-up. Hepatic and renal function were examined. The complete ablation, tumor volume shrinkage, and complication rates were analyzed.

**Results:**

The ablation time and energy of the 3D group were lower than those of the 2D group (1152.0 ± 403.9 s vs. 1379.7 ± 375.8 s and 87,407.2.9 ± 50,387.0 J vs. 117,775.8 ± 46,245.6 J, *P* = 0.031 and 0.021, respectively). The 3D group had a higher complete ablation rate than the 2D group (97.7 ± 2.4% vs. 94.5 ± 3.7%, *P* < 0.001). The incidence of hemoglobinuria after ablation in the 3D group was lower than that in the 2D group (32.0% vs. 57.6%, *P* = 0.047). The levels of alanine aminotransferase (ALT), aspartate aminotransferase (AST), alkaline phosphatase (ALP), and creatinine (Cre) after ablation in the 3D group were lower than those in the 2D group (126.7 ± 56.4 U/L vs. 210.9 ± 96.2 U/L, *P* < 0.001; 141.0 ± 60.8 U/L vs. 211.4 ± 90.0 U/L, *P* = 0.001; 57.3 ± 17.6 U/L vs. 80.8 ± 41.9 U/L, *P* = 0.010; and 66.6 ± 16.6 mmol/L vs. 84.5 ± 39.6 mmol/L, *P* = 0.037, respectively). There were no significant differences in antenna insertion and the volume reduction rate between the groups. One patient developed acute kidney injury shortly after ablation in the 2D group and recovered after hemodialysis. No other severe complications occurred during the follow-up period.

**Conclusions:**

The 3D visualization operative planning system has a relatively high clinical application value in providing scientific, reasonable, quantifiable, and individualized therapy for LHHs by US-PMWA.

## Background

Hepatic hemangioma is the most frequently encountered solid benign liver neoplasm, with an incidence of 0.4 to 20% in the general population and accounting for 73% of all benign liver lesions [1–3]. There is no controversy concerning the management of incidentally identified and asymptomatic hepatic hemangiomas, which require no intervention or further observation if there are no adverse complications or effects on adjacent organs. Only large hepatic hemangiomas (LHHs) that lead to symptoms such as abdominal pain or distension, anemia, portal hypertension, obstructive jaundice, spontaneous or traumatic rupture, coagulopathy or serious psychological effects may require intervention [4–9]. However, the definition of LHHs varies in the literature. Some authors report hemangioma > 4 cm as large [7, 10, 11], while a very small number of authors define those > 10 cm as LHH [12–14]. However, in most reports in the literature, LHHs are defined as being ≥5 cm and warranting therapy when they lead to clinical symptoms or the risk of life-threatening spontaneous rupture and hemorrhage [5, 7, 15]. In this study, LHH was defined as a hemangioma with a maximum diameter ≥ 5 cm.

Surgical resection and surgical enucleation are the traditional modalities for the treatment of LHHs [9, 13, 16–18]. However, other therapies, including liver transplantation, transcatheter arterial embolization [19–22], radiation therapy [23, 24], thermal ablation (radiofrequency and microwave ablation) [3, 8, 11, 25–27], and medications (bevacizumab, sorafenib, cisplatin and bleomycin) [28, 29], have also been reported in the management of LHHs. In some cases, operative treatment of LHHs remains a challenge for surgeons because of massive intraoperative blood loss and difficulty in controlling bleeding [30, 31]. Compared with other ablation technique, the benefits of microwave ablation, which has a higher thermal efficiency, include fewer limitations due to the heat-sink effect and tissue charring and the achievement of a larger ablation volume in a shorter time, with better clinical effects [11, 32–34]. Therefore, microwave ablation is a better choice for the treatment of symptomatic LHHs.

Importantly, LHHs often compress surrounding vital structures, such as the gastric outlet, hepatic vein, portal vein, hepatic artery, bile duct, or inferior vena cava, which could increase the difficulty and risk of treatment [35–37]. Methods to protect surrounding organs and simultaneously achieve complete ablation are important. Moreover, hemoglobinuria and acute renal failure occurring after ablation have been reported in the literature, including cases requiring hemodialysis [10, 38]. Therefore, how to achieve scientific, objective, quantifiable, and individualized operative planning is a key issue concerning ablation. Operative planning is typically performed based on the spatial awareness and experience of operators’ “human brain” using two-dimensional (2D) imaging data, which are highly subjective and abstract. The operators’ evaluation is inconsistent with the actual situation in some cases and may lead to incomplete ablation or major complications [39]. A three dimensional (3D) visualization operation planning system not only displays the location and spatial relationship of the tumor with the surrounding structures, quantifies the tumor size and volume, predicates the time-temperature profile, and improves the safety and effectiveness of ablation but also enables planning of the implantation route and accurate positioning of the ablation antenna [40–42]. The purpose of this study was to explore the clinical value of a 3D visualization operative planning system in ultrasound-guided percutaneous microwave ablation (US-PMWA) for large hepatic hemangiomas compared to the 2D operative planning system.

## Methods

### Patients and study design

This single-center, retrospective study protocol was approved by the Ethics Committee of the Chinese PLA General Hospital (Beijing, China) and was conducted in accordance with the principles of the Declaration of Helsinki. From January 2011 to August 2018, 58 patients (43 females, 15 males; mean age 43.5 ± 8.2 years) with 58 LHHs (mean maximum diameter 7.4 ± 1.5 cm, range 5–12.6 cm) were divided into two groups. The 3D group included 25 patients who underwent US-PMWA with the aid of the self-developed 3D visualization operative planning system. The 2D group included 33 patients who underwent US-PMWA using the conventional 2D image operative planning methods.

The inclusion criteria for US-PMWA were as follows: (1) definite diagnosis of LHH ≥5 cm based on the typical enhancement pattern on contrast-enhanced computed tomography (CT), magnetic resonance imaging (MRI), or contrast-enhanced ultrasound (CEUS); (2) clinical symptoms typically caused by LHHs present for at least 1 year, including abdominal pain, nausea, vomiting, abdominal fullness, and serious mental burden [10, 11]; (3) diagnoses of LHH pathologically proven by ultrasound (US)-guided core needle biopsy before ablation; and (4) prothrombin time < 25 s; prothrombin activity > 40%; and platelet count > 60 cells × 10^9^/L. The exclusion criteria were: (1) severe cardiopulmonary disease; (2) serious renal failure (creatinine above the normal level); (3) severe hepatic failure (aminotransferase and bilirubin greater than the twice the normal level). Written informed consent was obtained from all participating patients.

### 3D visualization operative planning system, US-PMWA, and auxiliary techniques

#### 3D visualization operative planning system

All patients with LHH underwent conventional ultrasound, CEUS, and/or CT/MRI to delineate the target tumor within 7 days before ablation. A desktop computer (Lenovo) with an Intel Core i5 processor for an empirical study in our department was used to perform 3D visualization preoperative planning. A series of CT or MRI data (0.625 mm- or 2.5 mm-thick slices) of the LHHs before MWA were converted to DICOM format and then imported into the 3D visualization treatment platform (Hokai Company, Zhuhai, China). Our group originally developed the 3D visualization platform software that has novel functions as follows: 1) rapid segmentation in the target (within 2 min); 2) volume calculation in the target; 3) simulation of the thermal field; 4) interactive pre-ablative planned strategy by manual operation; and 5) assessment of ablative effect by tumor mapping. The graphical user interface displayed the real-time simulation 2D ultrasound guided planning and the 3D visualization planning, as well as the planning path from the transverse, coronal, and sagittal planes. The application of 3D visualization technology enables a radiologist to perform various operations on the 3D image, such as free movement, rotation, and scaling, to develop a puncture plan by seeing more intuitively. The liver mass with a 5-mm tumor-free margin, vessels, and surrounding vital structures was segmented and reconstructed, which can be demonstrated via stereo display in the 3D visualization.

The 3D operation planning system of US-PMWA should abide by the following principles: (1) conformational ablation was applied while covering the entire tumor only; (2) avoid ablation of vital structures, particularly the secondary biliary duct, arteries and veins, or intestinal tract; and (3) minimize the distance of electrode insertion trajectories while avoiding puncture pathways via critical structures. The planning system was designed to iterate these goals until a reasonable and feasible plan was achieved [39].

### Us-PMWA

The ablations were performed in our institution, and all patients were hospitalized. The microwave system (KY-2000, Kangyou Medical, Nanjing, China) comprised a MW generator, flexible coaxial cable, and cooled-shaft antenna. A 2450 MHz or 915 MHz MW system was used. The 2450 MHz MW system consisted of three independent MW generators, three flexible coaxial cables, and three water-pumping machines that can simultaneously drive three 15-gauge cooled-shaft antennae (1.1 cm antenna tip). The 915 MHz MW system consisted of two independent MW generators, two flexible coaxial cables, and two water-pumping machines that can simultaneously drive two 15-gauge cooled-shaft antennae (2.2 cm antenna tip). The MW system generators were capable of producing 1-100 W of power output. The 15-G cooled-shaft antenna was coated with Teflon to prevent adhesion with dual channels inside the antenna shaft, through which distilled water continuously circulated via a peristaltic pump that could cool the shaft to prevent overheating.

The patient was unconscious, under intravenous anesthesia (propofol, 6-12 mg/kg/h; ketamine, 1-2 mg/kg), during ablation in the operating room. All treatments were carried out by two operators with more than 5 years of ablation experience according to the operative plan. If the tumors and tumor-feeding arteries were not well visualized with conventional US, CEUS-guided ablation with SonoVue (Bracco, Milan, Italy) was performed. US-guided biopsy was performed using an 18-G cutting needle (C. R. Bard, Japan) before ablation. First, the tumor-feeding artery of the hemangioma was ablated with higher power (60-80 W) until no blood flow or hyperenhancement was visible on color Doppler flow imaging (CDFI) and CEUS. An 18-G percutaneous transhepatic cholangiography (PTC) needle (Hakko, Japan) was inserted into the hemangioma to simultaneously aspirate the blood from the blood sinus of the hemangioma. Two antennae were subsequently implanted in the proper location of the LHH according to the 3D visualization operative planning system. The hyperechoic area of ablation was monitored by greyscale ultrasound, and the endpoint of ablation was determined. The antennae tracks were routinely cauterized to avoid bleeding during withdrawal.

### Auxiliary techniques

According to the preoperative contrast-enhanced CT or MRI imaging or 3D visualization operative planning system evaluation, hydrodissection and thermal monitoring techniques were applied for LHHs abutting vital structures to avoid thermal injury. After application of a local anesthetic (1% lidocaine), a 16-G intravenous catheter (BD Angiocath; Sandy, UT, USA) was inserted into the peritoneal cavity between the edge of the liver and the abutting gastrointestinal tract using US guidance. A sufficient amount of chilled normal saline was delivered until a separation of greater than 0.5 cm was achieved and maintained during the procedure. Then, 21-G thermocouple needles were equipped on the MWA system, which are easily visualized by US during ablation, for LHHs abutting vital structures. Based on our experimental evidence, the temperature cut-off of ablation was set at 60 °C. If the measured temperature reached 60 °C, ablation was immediately terminated and activated again after the temperature decreased to 50 °C [10, 11].

### Therapeutic effect evaluation and follow-up

**Fig. 1:**
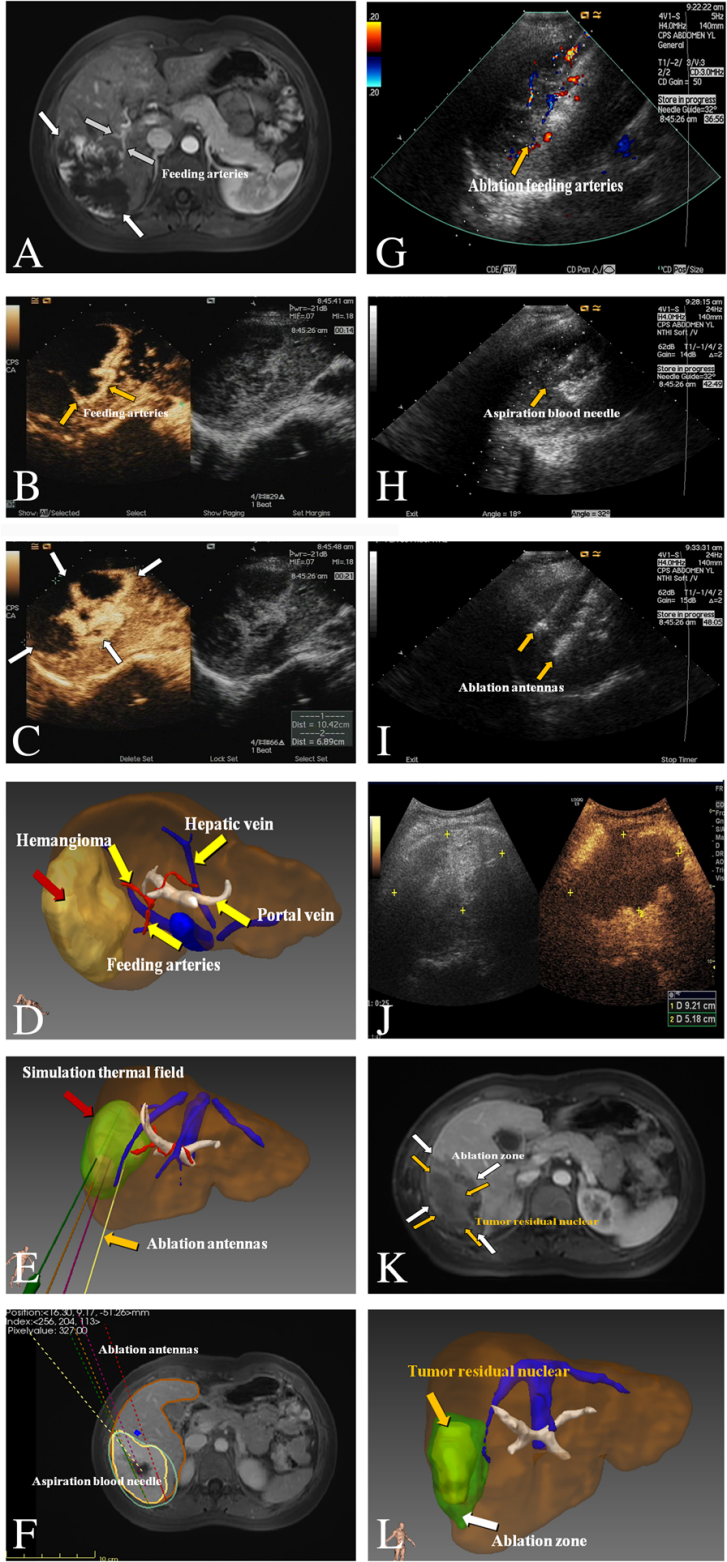
Images of a 28-year-old woman who underwent US-PMWA for larger hepatic cavernous hemangioma (LHH) (10.4 cmx6.9 cmx8.4 cm) assisted by a 3D visualization operative planning system. **a** Preoperative contrast-enhanced MRI showed the LHH with peripheral nodular hyperenhancement in the artery phase in the right lobe accompanied by two feeding arteries (white arrows). **b**-**c** Preoperative contrast-enhanced ultrasound imaging also showed the LHH with peripheral nodular hyperenhancement in the artery phase in the right lobe (white arrow) companying with two feeding arteries (yellow arrow). **d**-**f** 3D visualization operative planning system showed the location and relationship with the tumor and the surrounding organs and the feed arteries stereoscopically (yellow arrow), quantized the volume of the liver and LHH (liver: 1396.94 ml; LHH:197 ml), projected the number and the pathway of the ablation antenna implantation, the ablation time and energy, simulated the thermal field, and provided the location of the aspiration blood needle, which is at the center of the LHH. **g**-**i** The ablation procedure was performed according to the panning. First, the arteries were ablated by higher power (**g**) and blood was aspirated from the sinus (**h**). Two antennas were subsequently implanted in the LHH according to planning with two insertion and eight ablation points (**i**) (yellow arrow). **j**-**l** After ablation, the CEUS image immediately after ablation showed no enhancement in the ablation zone with a shrunk size (**j**). In the MRI image 3 days after ablation, the tumor residual nuclear and ablation zones were clearly demonstrated, and the residual nuclear zone had largely shrunk (**k**). The post-operative 3D visualization system safely showed the tumor residual nuclear and ablation zones and the surrounding organs; the volumes of the tumor residual nuclear and ablation zones and the reduction rate of the volume were also calculated (58.6 ml, 131.0 ml and 70.3%) (**l**) (yellow and white arrows)

The therapeutic effect was assessed by ultrasound at 1, 3, 6, and 12 months for 1 year and then at 6-month intervals after ablation during the follow-up period. If a recurrence tumor was detected by more than 40–50% of the ablation zone, contrast-enhanced imaging was performed. Technical effectiveness was defined as 90–100% of the hemangiomas ablated on contrast enhanced imaging within 3 days after ablation. The complete ablation was evaluated via an enhancement on contrast-enhanced imaging and calculated by the post 3D planning system (Fig. 1a-l). The complete ablation rate and tumor volume shrinkage rate were analyzed. Complications were classified according to the Society of Interventional Radiology Classification system for Complications by Outcome [43]. The hepatic and renal function and routine urine test results were examined before and 1 day after ablation. Once abnormal results were detected, medical treatment was provided until resolution occurred. The number of antenna insertions, tumor volume, ablation time, ablation energy, and aspiration blood volume were recorded and analyzed.

### Statistical analysis

Data were analyzed using SPSS 21.0 for Windows (SPSS Inc., Chicago, IL, USA). Continuous data are expressed as the mean ± standard deviation (SD). The paired t-test or x^2^ test was used to compare values between two groups. The comparison of continuous variables between two groups was performed via Student’s t-test or the Mann-Whitney U-test. Changes in the hepatic and renal function before and after ablation were compared using paired t-tests. Pearson chi-squared analysis or Fisher exact tests were performed to compare the categorical variables. *P* values < 0.05 were considered statistically significant.

## Results

### Basic characteristics of patients, tumors, and ablations

Table 1 reports all patient, LHH, and ablation data. Among 58 LHHs, 11 lesions were located in the left lobe compressing the gastric outlet; 47 lesions were located in the right lobe, with 10 lesions abutting the intestinal tract. Hydrodissection and thermal monitoring techniques were applied for 21 patients with a 100% one-time success rate. All patients successfully underwent a single ablation according to 2D or 3D pre-operative planning. The aspiration blood volume was 108.5 ± 79.2 ml (14-359 ml), the ablation energy was 104,685.9 ± 49,997.8 J (3000-226,400 J), the ablation time was 1281.6 ± 401.2 s (480–2230 s), the antenna insertion was 4.4 ± 1.6 (2–9), the complete ablation rate was 95.9 ± 3.5% (90–100%), and the reduction rate of the tumor volume was 52.1 ± 8.4% (35.2–76.7%).

**Table 1:** The characteristics of patients, tumors and ablations of LHHs patients

Parameters	Date
Patients (No.)	58
Male/Female (No.)	15/43
Age (years)	43.5±8.2(28-60)
Tumors (No.)	58
Mean max diameter (cm)	7.4±1.5(5-12.6)
Volume of tumor (ml)	136.9±79.4(26.8-375.9)
Location (right/left)	47/11
Hydrodissection (ml)	21
Thermal monitoring	21
Aspiration blood volume (ml)	108.5±79.2(14-359)
Ablation energy (J)	104685.9±49997.8(3000-226400)
Ablation time (s)	1281.6±401.2(480-2230)
Antenna insertion (No.)	4.4±1.6(2-9)
Complete ablation rate (%)	95.9±3.5(90-100)
Reduction rate of volume (%)	52.1±8.4(35.2-76.7)
Rate of hemoglobinuria (%)	46.5(27/58)

### Changes in hepatic and renal function before and after ablation

The preoperative characteristics and postoperative findings are reported in Table 2. The values of alanine aminotransferase (ALT), aspartate aminotransferase (AST), total bilirubin (TB), direct bilirubin (DBIL), and creatinine (Cre) after ablation were higher than the values before ablation, with significant differences ((22.8 ± 16.9 U/L (6.8–84.1) vs. 174.6 ± 91.1 U/L (37–543.9), *P* < 0.001; 19.0 ± 7.0 U/L (10.6–40.0) vs. 181.0 ± 85.7 U/L (32.0–479.9), *P* < 0.001; 12.3 ± 4.6 U/L (5.4–26.6) vs. 30.5 ± 14.3 U/L (3.8–74.1), *P* < 0.001; 4.9 ± 1.4 U/L (2.3–7.8) vs. 9.6 ± 8.1 U/L (1.2–59.2), *P* < 0.001; and 59.6 ± 13.0 mmol/L (34.5–92.9) vs. 76.1 ± 32.7 mmol/L (38.2–204.0), *P* = 0.010, respectively). Although the indexes of alkaline phosphatase (ALP), gamma-glutamyl transpeptidase (GGT), and blood urea nitrogen (BUN) after ablation were higher than the values before ablation, no significant differences were identified (64.3 ± 16.5 U/L (39.4–98.8) vs. 70.7 ± 35.4 U/L (19.4–178.9), *P* = 0.179; 24.2 ± 13.6 U/L(6.7–80.2) vs. 33.0 ± 19.2 U/L (1.9–92.8), *P* = 0.150; and 4.5 ± 1.2 μmol/L (2.6–7.9) vs. 5.6 ± 4.5 μmol/L (1.3–26.4), *P* = 0.146, respectively). All data suggested that the effects of US-PMWA for LHH on hepatic and renal functions were transient and resolved rapidly with medical treatment. While the incidence rate of hemoglobinuria was 46.5% (27/58), only 1 patient developed acute renal failure; this patient recovered after hemodialysis. No other severe complications occurred during the perioperative and follow-up periods.

**Table 2:** The changes of liver and renal function before and after ablation in LHHs patients

Parameters	Pre-operative	Post-operative	*P* value
ALT(U/L)	22.8±16.9(6.8-84.1)	174.6±91.1(37-543.9)	<0.001*
AST(U/L)	19.0±7.0(10.6-40.0)	181.0±85.7(32.0-479.9)	<0.001*
ALP (U/L)	64.3±16.5(39.4-98.8)	70.7±35.4(19.4-178.9)	0.179
GGT (U/L)	24.2±13.6(6.7-80.2)	33.0±19.2(1.9-92.8)	0.150
Cre (umol/L)	59.6±13.0(34.5-92.9)	76.1±32.7(38.2-204.0)	0.010*
BUN (mmol/L)	4.5±1.2(2.6-7.9)	5.6±4.5(1.3-26.4)	0.146
STB (U/L)	12.3±4.6(5.4-26.6)	30.5±14.3(3.8-74.1)	<0.001*
DBIL (U/L)	4.9±1.4(2.3-7.8)	9.6±8.1(1.2-59.2)	<0.001*

*ALT* alanine aminotransferase, *AST* aspartate aminotransferase, *ALP* alkaline phosphatase, *GGT* gamma-glutamyl transpeptidase, *Cre* creatinine, *BUN* blood urea nitrogen, *STB* total bilirubin, *DBIL* direct bilirubin; *-pre: index of preablation; *-post: index of postablation

### Comparison of clinical characteristics between the 3D planning group and 2D planning group

The characteristics of the 3D and 2D groups before ablation are shown in Table 3. There were no significant statistical differences between the groups in terms of age, mean maximal tumor diameter, tumor volume, and aspiration blood volume (43.8 ± 8.8 years (28–57) vs. 43.2 ± 7.9 years (31–60); 7.5 ± 1.7 cm (5–12.5) vs. 7.2 ± 1.3 cm (5.1–10.8); 148.2 ± 89.1 ml (26.8–301.1) vs. 128.4 ± 71.4 ml (39.8–375.8); and 108.4 ± 84.9 ml (14–284) vs. 108.5 ± 75.9 ml (21–359); *P* = 0.766, 0.451, 0.352, and 0.996, respectively). No significant differences were identified in hepatic and renal function as measured by ALT, AST, ALP, GGT, Cre, BUN, STB, and DBIL before ablation (20.3 ± 15.2 U/L (6.9–84.0) vs. 24.7 ± 18.0 U/L (9.0–107.5); 19.2 ± 6.5 U/L (11.8–38.6) vs. 18.9 ± 7.4 U/L (10.8–34.6); 63.0 ± 18.1 U/L (40.4–98.8) vs. 65.2 ± 15.4 U/L (39.8–94.3); 23.5 ± 16.6 mmol/L (8.6–38.1) vs. 24.8 ± 11.0 mmol/L (6.7–48.2); 63.0 ± 8.9 μmol/L (49.9–78.7) vs. 57.2 ± 15.1 μmol/L (37.6–85.9); 4.7 ± 1.2 mmol/L (2.8–7.8) vs. 4.2 ± 1.0 mmol/L (2.6–6.4); 12.3 ± 3.6 U/L (6.4–20.1) vs. 12.4 ± 5.3 U/L (5.4–26.3); and 4.5 ± 1.3 U/L (2.3–7.5) vs. 5.2 ± 1.5 U/L (2.9–7.9); *P* = 0.330, 0.872, 0.618, 0.709, 0.092, 0.055, 0.923, and 0.070, respectively). Thus, the baseline data of the two groups showed no differences, and the outcomes were comparable.

**Table 3:** The comparison of clinical parameters between 3D planning group and 2D planning group group patients in LHHs patients

Parameters	3D planning group	2D planning group	*P* value
Age (years)	43.8±8.8(28-57)	43.2±7.9(31-60)	0.766
Mean max diameter (cm)	7.5±1.7(5-12.5)	7.2±1.3(5.1-10.8)	0.451
Volume of tumor (ml)	148.2±89.1 (26.8-301.1)	128.4±71.4(39.8-375.8)	0.352
Aspiration blood volume (ml)	108.4±84.9 (14-284)	108.5±75.9(21-359)	0.996
Ablation power (J)	87407.2.9±50387.0(3000-226400)	117775.8±46245.6(6000-200800)	0.021*
Ablation time (s)	1152.0±403.9(600-1890)	1379.7±375.8(810-2230)	0.031*
Antenna insertion	4.8±1.9(2-9)	4.1±1.2(2-6)	0.068
Complete ablation rate (%)	97.7±2.4(93-100)	94.5±3.7(90-100)	0.0004*
Reduction rate of volume (%)	53.4±7.5(43.5-76.0)	51.1±9.0(35.2-76.7)	0.299
Case of hemoglobinuria (No.)	8/17	19/14	0.047*
ALT-pre(U/L)	20.3±15.2(6.9-84.0)	24.7±18.0(9.0-107.5)	0.330
AST-pre(U/L)	19.2±6.5(11.8-38.6)	18.9±7.4(10.8-34.6)	0.872
ALP-pre(U/L)	63.0±18.1(40.4-98.8)	65.2±15.4(39.8-94.3)	0.618
GGT-pre(U/L)	23.5±16.6(8.6-38.1)	24.8±11.0(6.7-48.2)	0.709
Cre-pre(umol/L)	63.0±8.9 (49.9-78.7)	57.2±15.1(37.6-85.9)	0.092
BUN-pre(mmol/L)	4.7±1.2(2.8-7.8)	4.2±1.0(2.6-6.4)	0.055
STB-pre (U/L)	12.3±3.6(6.4-20.1)	12.4±5.3(5.4-26.3)	0.923
DBIL-pre (U/L)	4.5±1.3(2.3-7.5)	5.2±1.5(2.9-7.9)	0.070
ALT-post (U/L)	126.7±56.4(37.0-224.1)	210.9±96.2(94.8-345.0)	0.0002*
AST-post (U/L)	141.0±60.8(40.2-237.0)	211.4±90.0(32-294.5)	0.001*
ALP-post(U/L)	57.3±17.6(34.6-79.4)	80.8±41.9(36.4-178.0)	0.010*
GGT-post(U/L)	26.5±17.6(9.4-64.2)	37.8±19.1(8.8-92.8)	0.060
Cre-post(umol/L)	66.6±16.6(51.3-117.0)	84.5±39.6(40.8-204.0)	0.037*
BUN-post(mmol/L)	4.4±2.6(1.3-13.8)	6.6±5.3(2.4-18.1)	0.061
STB-post (U/L)	28.0±14.4(8.7-70.7)	32.4±14.2(3.8-74.1)	0.252
DBIL-post (U/L)	8.4±4.6 (1.2-17.7)	10.6±10.0(3.4-49.4)	0.428

*ALT* alanine aminotransferase, *AST* aspartate aminotransferase, *ALP* alkaline phosphatase, *GGT* gamma-glutamyl transpeptidase, *Cre* creatinine, *BUN* blood urea nitrogen, *STB* total bilirubin, *DBIL* direct bilirubin; *-pre: index of preablation; *-post: index of postablation

Table 3 reports the results after ablation in the 3D and 2D groups. The ablation time and energy of the 3D group were lower than those of the 2D group (1152.0 ± 403.9 s (600–1890) vs. 1379.7 ± 375.8 s (810–2230) and 87,407.2.9 ± 50,387.0 J (3000–226,400) vs. 117,775.8 ± 46,245.6 J (6000–200,800); *P* = 0.031 and 0.021, respectively). The 3D group had a higher complete ablation rate than that of the 2D group (97.7 ± 2.4% (93–100) vs. 94.5 ± 3.7% (90–100), *P* < 0.001). The incidence of hemoglobinuria after ablation in the 3D group was lower than that of the 2D group (32.0% vs. 57.6%, *P* = 0.047). The hepatic function indices of ALT, AST, and ALP and the renal function index of Cre in the 3D group after ablation were lower than those of the 2D group (126.7 ± 56.4 U/L (37.0–224.1) vs. 210.9 ± 96.2 U/L (94.8–345.0), *P* < 0.001; 141.0 ± 60.8 U/L (40.2–237.0) vs. 211.4 ± 90.0 U/L (32–294.5), *P* = 0.001; 57.3 ± 17.6 U/L (34.6–79.4) vs. 80.8 ± 41.9 U/L (36.4–178.0), *P* = 0.010; and 66.6 ± 16.6 μmol/L (51.3–117.0) vs. 84.5 ± 39.6 μmol/L (40.8–204.0), *P* = 0.037, respectively). There were trends towards differences in the antenna insertion and tumor volume reduction rate between the groups, without significant differences (4.8 ± 1.9 (2–9) vs. 4.1 ± 1.2 (2–6); 53.4 ± 7.5% (43.5–76.0) vs. 51.1 ± 9.0% (35.2–76.7); *P* = 0.068 and 0.299, respectively). The increases in GGT, BUN, STB, and DBIL in the 3D group were slightly lower than those in the 2D group, without significant statistical differences (26.5 ± 17.6 U/L (9.4–64.2) vs. 37.8 ± 19.1 U/L (8.8–92.8), 4.4 ± 2.6 mmol/L (1.3–13.8) vs. 6.6 ± 5.3 mmol/L (2.4–18.1); 28.0 ± 14.4 U/L (8.7–70.7) vs. 32.4 ± 14.2 U/L (3.8–74.1); 8.4 ± 4.6 U/L (1.2–17.7) vs. 10.6 ± 10.0 U/L (3.4–49.4); *P* = 0.060, 0.061, 0.252, and 0.428, respectively). One patient developed a case of acute kidney injury shortly after ablation in the 2D group and recovered after 12 hemodialysis episodes and 1 month of medical treatment.

## Discussion

Concerning the treatment of symptomatic LHHs, a benign neoplasm, the primary goal is to relieve clinical symptoms and reduce the risk of rupture and bleeding. There are many therapeutic options, including surgical resection, transcatheter arterial embolization, thermal ablation, steroid treatment, radiation therapy, hepatic arterial ligation, and thermal ablation. Image-guided thermal ablation has been broadly applied clinically due to its advantages of minimal invasion, safety, convenience, efficacy, tolerability, and a shorter recovery time. Hepatic hemangiomas are benign tumors of the liver that consist of clusters of blood-filled cavities (sinusoids), lined by endothelial cells and fed by the hepatic artery [44].

The typical cause of hemangioma formation is abnormal vascular development during embryonic development due to a lack of smooth muscle tissue in abnormal blood vessels, leading to slow blood flow, blood stagnation, and slow heat dissipation [1]. MWA has the advantages of a broader thermal range and a more even spread, rapid heating speed, and reduced influence of carbonization and blood-flow perfusion; furthermore, multiple antennas can conduct ablation simultaneously without interference, exerting a synergistic effect, thus achieving a broader ablation range and shorter ablation time. Thus, MWA is the most suitable choice [11, 45].

The complex association between LHH and the surrounding vital structures could increase the difficulty and risk of treatment, prolong the operation time, increase ablation energy requirements, and potentially lead to damage to veins, bile ducts, or the intestinal tract and incomplete ablation [46–50]. It has been reported, based on a multivariate analysis, that the ablation time was an independent risk factor associated with hemoglobinuria [10, 45]. In this comparative study, the data showed that the ablation time and energy of the 3D group were lower than those of the 2D group. The 3D group had a higher complete ablation rate than the 2D group. The incidence of hemoglobinuria after ablation in the 3D group was lower than that of the 2D group. The effects on hepatic and renal function in the 3D group were milder than those in the 2D group. These results indicate that the 3D preoperative planning system provided valuable anatomical information regarding the LHHs and surrounding organs, displayed stereoscopically.

The calculation of the tumor volume, programming of the antenna implantation, and precise prediction of the thermal field were also well planned, enabling a safe, precise, and successful ablation. The influence on renal function was reduced and the incidence of hemoglobinuria was lower with the 3D visualization operative planning system. The results indicated that the LHHs were ablated, rather than normal liver tissue. Conformal ablation was achieved with combination therapy. There was no thermal damage to the veins, bile ducts, and intestinal tract attributed to the auxiliary techniques, such as hydrodissection and thermal monitoring techniques [11, 51].

In ablation therapy, the tumor volume reduction rate is primarily dependent on the percentage of tumor ablation. The edge of LHHs is moved farther from high risk organs due to volume reduction after the ablation process, which leads to the relief of clinical symptoms. While incomplete ablation can occur and hepatic and renal function can be markedly affected, “eccentric ablation therapy” can be achieved in some cases. Although no significant differences were identified, the volume reduction rate of the 3D visualization operative planning group was slightly higher. These data further suggest the importance of the 3D visualization operative planning system in LHH ablation.

Serious complications should not occur in the treatment of benign lesions, including LHHs. One case of acute kidney injury shortly after ablation, with recovery after 12 hemodialysis treatments and 1 month of medical treatment, occurred in the 2D group. For this patient, the LHH maximum diameter was 8.6 cm and the volume was 295.29 ml, which was not the largest LHH included in this study. The ablation time was 1980 s and the ablation energy was 326,400 J; both of these variables are risk factors for complications. The ALT, AST, and Cre levels after ablation were 489 U/L, 1063.1 U/L, and 227 μmol/L, respectively, which were markedly increased from the pre-treatment values (17.3 U/L, 20.8 U/L, and 75.3 μmol/L, respectively), which indicates that more liver tissue was ablated, as verified by imaging. However, more treatments administered to patients may increase mental stress. Scientific, objective, quantifiable, and individualized operative planning are key issues for safe and effective ablation.

Although this study was a comparative study, there are several limitations. First, this study was a retrospective investigation; thus, a prospective, randomized, controlled trial would provide more scientifically valid data. Second, the study was performed in a single institution with a relatively small sample. The results should be confirmed by another cohort or a prospective, multicenter study with a larger sample size. Third, with the development and incorporation of applied mathematics, physics, and medicine, a more quantifiable, predictable, and controllable 3D visualization operative planning system may be developed that will provide more benefits for patients and operators.

## Conclusion

The 3D visualization operation planing system provided more spatial imaging information and data on the relationship of the tumor with the surrounding structures; it also quantified the ablation techniques and the thermal field, providing scientific, objective, reasonable, quantifiable, and individualized operative planning for US-PMWA of LHHs. These factors are key issues for safe and effective ablation. Compared with the 2D group, the 3D group showed a higher complete ablation rate and a comparable reduction volume rate, with less effects on hepatic and renal function and a lower incidence of hemoglobinuria. Therefore, the 3D visualization operative treatment planning system has a relatively high clinical application value for use with US-PMWA for LHHs.

## Abbreviations

2D: Two-dimensional
3D: Three dimensional
ALP: Alkaline phosphatase
ALT: Alanine aminotransferase
AST: Aspartate aminotransferase
BUN: Blood urea nitrogen
CDFI: Color Doppler flow imaging
CEUS: Contrast-enhanced ultrasound
Cre: Creatinine
CT: Computed tomography
DBIL: Direct bilirubin
GGT: Gamma-glutamyl transpeptidase
LHHs: Large hepatic hemangiomas
MRI: Magnetic resonance imaging
PTC: Percutaneous transhepatic cholangiography
SD: Standard deviation
TB: Total bilirubin
US: Ultrasound
US-PMWA: Ultrasound-guided percutaneous microwave ablation
